# Multimodal Targeted Deep Sequencing of Circulating Tumor Cells and Matched Cell-Free DNA Provides a More Comprehensive Tool to Identify Therapeutic Targets in Metastatic Breast Cancer Patients

**DOI:** 10.3390/cancers12051084

**Published:** 2020-04-27

**Authors:** Corinna Keup, Markus Storbeck, Siegfried Hauch, Peter Hahn, Markus Sprenger-Haussels, Oliver Hoffmann, Rainer Kimmig, Sabine Kasimir-Bauer

**Affiliations:** 1 Department of Gynecology and Obstetrics, University Hospital of Essen, 45122 Essen, Germany; 2 QIAGEN, 40724 Hilden, Germany

**Keywords:** liquid biopsy, circulating tumor cells, cell-free DNA, circulating tumor DNA, next-generation sequencing, unique molecular indices, metastatic breast cancer patients, multi-parametric, multimodal, therapy stratification

## Abstract

Cell-free DNA (cfDNA) and circulating tumor cells (CTCs) exhibit great potential for therapy management in oncology. We aimed to establish a multimodal liquid biopsy strategy that is usable with minimized blood volume to deconvolute the genomic complexity of metastatic breast cancer. CTCs were isolated from 10ml blood of 18 hormone receptor-positive and human epidermal growth factor receptor 2-negative (HER2-) metastatic breast cancer patients. cfDNA was isolated from plasma generated after CTC depletion and targeted sequencing analyses were conducted. *PIK3CA* and *ESR1* variants were less common in CTC gDNA, while *ERBB2* variants were only detected in CTC gDNA. A total of 62% of all cfDNA variants were recovered in the matched CTC gDNA, while 72% of all variants were unique in either cfDNA (14 variants) or CTC gDNA (104 variants). The percentage of patients with no detectable cfDNA variants or CTC gDNA variants was 17%/11%, but a combined analysis identified variants in 94% of all patients. In univariate and multivariate regression models, *ESR1* variants in cfDNA and CTC gDNA correlated significantly with survival. We suggest a coordinated analysis of both fractions in order to provide a comprehensive genomic footprint that may contribute to identifying the most suitable therapy for each individual.

## 1. Introduction

In oncology, cell-free DNA (cfDNA) and, specifically, cell-free tumor DNA (ctDNA), defined by the presence of variants, as well as circulating tumor cells (CTCs), are liquid biopsy analytes displaying vast potential for therapy management due to their valuable information about tumor heterogeneity and clonal evolution [1].

High levels of ctDNA have been associated with a poor overall survival (OS) and increased tumor burden [2,3]. *ESR1* variants in cfDNA were specifically correlated with a shorter duration of endocrine treatment effectiveness in metastatic breast cancer (BC) patients [4]. Recently, *PIK3CA* variant detection in cfDNA was established as a companion diagnostic in clinical practice for hormone receptor-positive (HR+), human epidermal growth factor receptor 2-negative (HER2-) metastatic breast cancer (MBC) patients by FDA approval of the selective PI3Kα inhibitor Alpelisib for patients presenting *PIK3CA* variants in tumor tissue or plasma. cfDNA can be obtained without prior enrichment [5] and cfDNA assays have a high sensitivity as well as reproducibility [6]. However, within the cfDNA in its entirety, the ctDNA fraction is small [5]. 

The undisputable prognostic value of CTC enumeration in MBC was already shown 15 years ago [7] and was confirmed in large meta studies [8]. The enormous advantage of CTCs is the opportunity to analyze genomic, transcriptomic and proteomic parameters [5]. Regarding the mutational analysis of CTCs, the minimal number of CTCs and the consequently marginal DNA yield [9] led to the integration of whole-genome amplification (WGA) before sequencing [10,11,12,13,14]. Interestingly, *ESR1* variants were detected in CTCs and might indicate the impaired effect of aromatase inhibitor treatment in MBC patients [11]. CTCs are viable cells actively migrating into the circulation as potential seeds of metastasis [5], while cfDNA is mostly generated by necrosis and apoptosis and therefore might, instead, present dying cells [15]. Importantly, the differences between cfDNA and CTCs can be regarded as a chance for comprehensive real-time disease profiling from the same patient material. Interestingly, a mutational analysis of cfDNA and transcriptional analysis of CTCs using both analytes in parallel from matched minimized blood volume revealed synergistic information [16].

Only a few comparison studies characterizing cfDNA and CTCs have been published, but cfDNA and CTCs were mostly either isolated from samples taken at different time points [17] or from blood samples drawn into different preservative blood tubes [9,18,19]. For some studies, the isolation and molecular characterization of cfDNA and CTCs from matched EDTA blood samples were described, but the required blood volume was around 20 ml [20,21]. For appropriate comparability, it would be desirable to use the same blood sample with a minimized volume, drawn and stored under the same conditions for the isolation of both analytes in order to reach an unbiased comprehensive liquid biopsy in an “all from one tube” format.

Here, we elucidate the value of cfDNA and CTC variants by a) establishing a workflow for the isolation and sequencing of genomic DNA from CTCs (CTC gDNA) without WGA and b) comparing variants of the same deep-sequencing approach with specificity-guaranteeing unique molecular indices (UMIs) in matched cfDNA and CTC gDNA samples isolated from a minimized blood volume of a stringent HR+HER2- MBC cohort.

## 2. Results

### 2.1. Establishment of an Isolation Protocol for CTC gDNA

In several pre-experiments, published protocols for single cell gDNA isolation [9,22] and the combination of lysis of CTCs by AdnaLysisbuffer (included in the AdnaTest EMT-2/StemCell Select^TM^, QIAGEN) and purification using columns or beads, as well as a combination of these and other isolation and purification methods with whole-genome amplification (WGA; REPLI-G Single Cell (QIAGEN) [23]) were examined, but resulted in highly variable data.

Finally, the buffer chemistry for the use of the AdnaLysisbuffer, in combination with the QIAamp MinElute column, now called the AllPrep DNA/RNA Nano Kit prototype (QIAGEN), was optimized via the implementation of proteinase K incubation and the adjustment of the binding and washing conditions. The mean DNA yield isolated with this protocol and quantified by Qubit was 5.5 ng. WGA was excluded from the protocol. Subsequently, pyrosequencing successfully detected *PIK3CA* E454K in the samples with 10–200 spiked-in MCF7 cells in 5ml blood of healthy donor controls (Figure S1A). Targeted next-generation sequencing (NGS) on the MiSeq Sequencer (mean read fragments total (million): 0.6) with UMI integration (mean reads/UMI: 3.9) revealed the successful detection of the MCF7-specific variant *PIK3CA* E454K in the samples with 100 and 20 cells spiked into healthy donor blood (Figure S1A). NGS on the NextSeq Sequencer (mean read fragments total (million): 2.6) with UMI integration (mean reads/UMI: 10.3) detected the variant in samples with 100, 50 and 20 spiked cells (Figure S1), but the call quality of the variants was <20. It is important to mention that pyrosequencing and NGS revealed comparable allele frequencies of *PIK3CA* E454K (Figure S1A).

### 2.2. Sequencing Qualities

**Figure 1:**
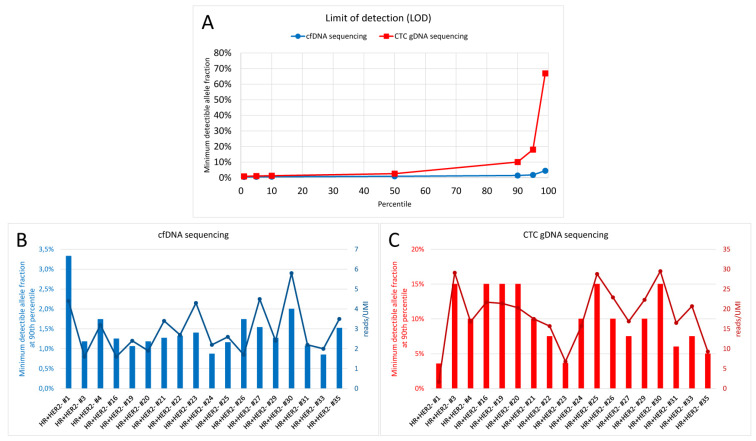
Limit of detection of the cfDNA and circulating tumor cell (CTC) gDNA sequencing analysis. (**A**) Lowest detectable variant allele frequency called with a confidence of 0–99% is plotted for cfDNA (blue) and CTC gDNA (red). The lowest detectable variant allele frequency called with 90% confidence is plotted for each of the 18 patient samples in comparison to the mean number of reads per UMI in the sample for cfDNA (**B**) and CTC gDNA (**C**).

The successful variant detection with minimally 20 cells in pre-experiments paved the way for CTC gDNA variant detection in 18 HR+ HER2- MBC patients with progressive disease. Additionally, a cfDNA variant analysis was conducted in matched samples. The comparison of the sequencing parameters of the two sequencing analyses (Table S2) revealed that, despite the high sequencing depth, the mean number of UMIs in each CTC gDNA sample was half of the mean number of UMIs in the cfDNA samples. Consequently, the mean UMI coverage was also only a half in the CTC gDNA samples compared with the cfDNA samples, while the read fragments per UMI were increased six-fold. Therefore, the lowest detectable variant allele frequency (VAF) called with a confidence of 90% (90th percentile estimated minimum detectible allele fraction) was dramatically increased in CTC gDNA samples (10%) compared with cfDNA samples (1%) (Table S2). Additionally, plotting the lowest detectable VAF called with a confidence of 0–99% allowed us to visualize a clear difference between the confidence of variant calling in cfDNA versus CTC gDNA, which was correlated with the number of reads per UMI (Figure 1).

### 2.3. Cohort

Of in total 18 included patients, 17 patients were older than 50 years when blood was drawn. The progress was documented, mostly for metastases in bone, liver and lymph nodes. The majority of patients presented at the time of first diagnosis with ductal, histological grade 2, T2 primary tumors with N1 status. At the time point of the last follow-up in December 2018, seven patients were alive, while eleven patients were deceased, with a median follow up time of 136 months (Table S1). We analyzed matched cfDNA and CTC gDNA samples from 28 patients, but the data of *n* = 10 patients were excluded due to an insufficient cfDNA library yield (*n* = 1) and a poor limit of detection for CTC gDNA variants (*n* = 9).

### 2.4. Patient-Specific Variants

After stringent variant filtering via QIAGEN GeneGlobe and Ingenuity Variant Analysis (IVA), 37 variants were detected in the cfDNA samples and 127 variants were detected in the matched CTC gDNA samples from 18 patients, respectively. The majority of cfDNA/CTC gDNA variants (86%/78%) were exclusively found in only one individual.

### 2.5. Localization of the Variants

**Figure 2:**
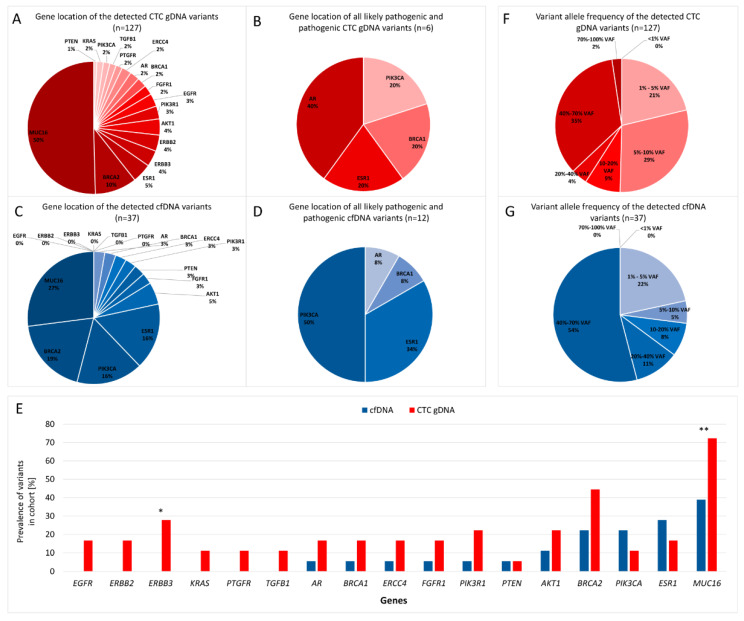
Distribution of the detected variants across the sequenced genes and their allele frequencies. **A**) Gene location of CTC gDNA variants, **C**) gene location of cfDNA variants. Distribution of pathogenic and likely pathogenic CTC gDNA (**B**) and cfDNA (**D**) variants across the sequenced genes. **E**) Prevalence of variants located within the same gene. Prevalence was calculated for the cohort of 18 hormone receptor-positive (HR+), human epidermal growth factor receptor 2-negative (HER2-) (HR+HER2-) metastatic breast cancer (MBC) patients. Potential differences between the prevalence of variants within identical genes in both fractions were calculated by exact two-sided Mann–Whitney U test (* = *p*-value <0.05; ** = *p*-value <0.005). Allele frequencies of the CTC gDNA (**F**) and cfDNA (**G**) variants.

Most variants were found in the *MUC16* gene in both analytes (Figure 2A,C). *BRCA2* variants were the second most prevalent variants in cfDNA and CTC gDNA. *PIK3CA* and *ESR1* variants were less common in CTC gDNA compared with cfDNA. This finding was also mirrored in the gene location of only the likely pathogenic or pathogenic variants (Figure 2B,D). In the CTC gDNA fraction, the most commonly detected likely pathogenic and pathogenic variants were located in the *AR* gene and the sum of the likely pathogenic and pathogenic *PIK3CA* and *ESR1* variants accounted for 40% of the variants (Figure 2B). In contrast, 84% of all likely pathogenic and pathogenic cfDNA variants were located in the *PIK3CA* and *ESR1* genes (Figure 2D).

### 2.6. Prevalence of Variants

**Figure 3:**
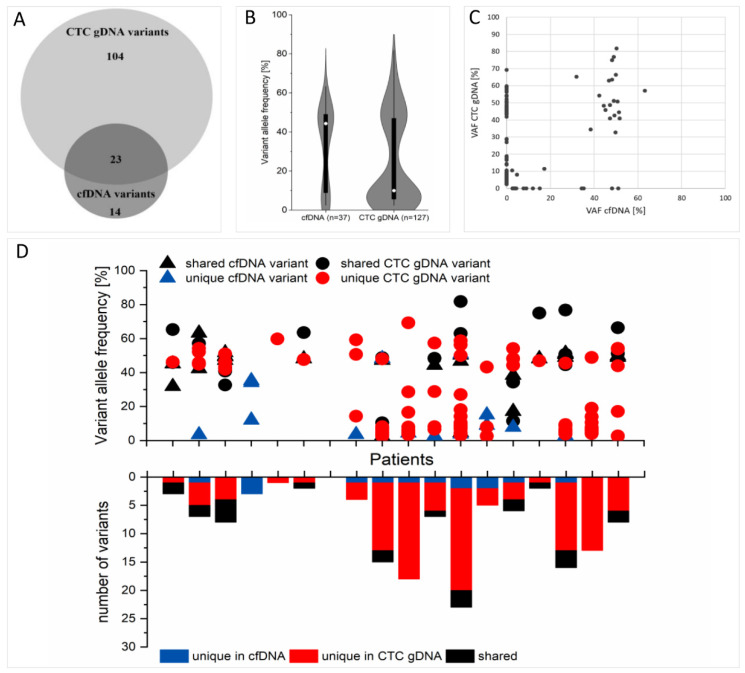
Direct comparison of cfDNA and CTC gDNA variants. (**A**) Venn diagram illustrating the concordance of detected cfDNA and CTC gDNA variants in 18 matched samples. (**B**) Violin diagram showing the distribution of variant allele frequencies of the detected variants in each fraction. The midpoints indicate the median, the black boxes indicate data from the first to the third quartile, the whiskers indicate the area within 1.5 of the interquartile range and the violins extend to the full range of data. (**C**) Variant allele frequencies (VAFs) of the unique and shared variants are plotted. (**D**) Top: VAFs of variants separated by patient and fraction. Bottom: number of variants separated by patients and fractions. Colors indicate whether the variants were found in cfDNA only (blue), CTC gDNA only (red) or whether the same variant was found in both cfDNA and CTC gDNA (black).

No cfDNA variant was detected in 3/18 patients, while no CTC gDNA variant was called in 2/18 patients (Figure 3D). Combined cfDNA and CTC gDNA sequencing revealed variants in 94% (17/18) of all patients. The mean number of CTC gDNA variants per patient was seven, while the cfDNA analysis revealed a mean of two variants per patient (Figure 3D).

In contrast to the absence of *EGFR, ERBB2, ERBB3, KRAS, PTGFR* and *TGFB1* variants in cfDNA, variants in all sequenced genes were detected in CTC gDNA, with a prevalence of at least 5% (Figure 2E). A relatively high prevalence was examined for *ESR1, BRCA2* and *MUC16* variants in both fractions. *PIK3CA* variants were detected in 22%/11% of all patients in the cfDNA and CTC gDNA fraction, while *ERBB2* variants were only detected with a prevalence of 28% in CTC gDNA. *MUC16* variants were called in 39%/72% of all patients in cfDNA/CTC gDNA and *ESR1* variants in 28%/17% of patients, respectively (Figure 2E). Thus, *PIK3CA* and *ESR1* variants were more prevalent in cfDNA compared to matched CTC gDNA. The prevalence of *MUC16* and *ERBB3* variants was significantly greater in the CTC gDNA fraction compared to the cfDNA fraction (calculated by a Mann–Whitney U test; *p*-value = 0.002 and *p*-value = 0.045). 

### 2.7. Variant Allele Frequency of the Variants

In cfDNA and CTC gDNA, no variant with a VAF of <1% was called. Approximately one quarter of all variants exhibited an allele frequency between 1–5% in cfDNA and CTC gDNA (Figure 2F,G and Figure 3B,C). In comparison to cfDNA, the fraction of CTC gDNA variants with VAF between 5–20% was increased threefold (Figure 2F,G).

### 2.8. Concordance of cfDNA and CTC gDNA Variants

A direct comparison of the individual variants in the respective patients revealed 14 unique cfDNA variants and 104 unique CTC gDNA. A total of 23 variants were identical in matched cfDNA and CTC gDNA, which is 28% of the entirety of the detected variants in both fractions (Figure 3A). The corresponding kappa value describing the overlap (Figure 3A) is -0.212, with a standard error of 0.057. Regarding the recovery of cfDNA variants in the CTC gDNA fraction, the shared variants represented 62% of all cfDNA variants (23/37). The mean VAF of all 23 shared variants was 42% in the cfDNA fraction and 48% in the CTC gDNA fraction, whereas variants exclusively found in cfDNA displayed a mean VAF of 17% and the unique CTC gDNA variants had a mean VAF of 20% (Figure 3B–D). Of the 72% of variants uniquely detected in one fraction, *PIK3CA* E726K, *PIK3CA* H1047R and *PIK3CA* N107S were only found in cfDNA, but not in CTC gDNA.

### 2.9. Prognostic Value

**Figure 4:**
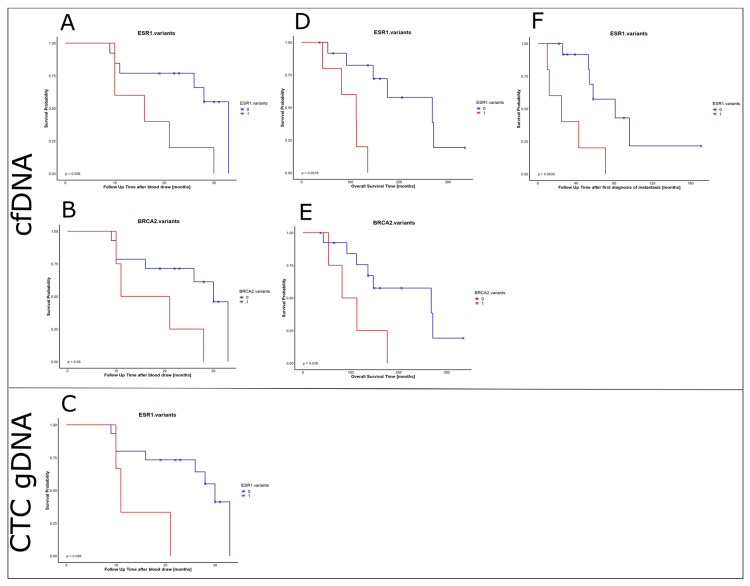
Kaplan Meier curves illustrating the prognostic value of *ESR1* variants and *BRCA2* variants. Data of patients with at least one variant located in the *ESR1* (**A**,**C**,**D**,**F**) or *BRCA2* (**B**,**E**) gene is depicted in red. Correlations were calculated to survival time after the blood was drawn (**A**–**C**), survival time after first diagnosis (**D**,**E**) and survival time after first diagnosis of metastasis (**F**). Only significant correlations (calculated by Log Rank (Mantel–Cox) test (*p*-value < 0.05)) are shown for cfDNA variants (**A**,**B**,**D**–**F**) and CTC gDNA (**C**).

The presence of variants within the 17 different sequenced genes was correlated with the survival parameters of the HR+HER2- MBC patients. *ESR1* variants in both, cfDNA and CTC gDNA, significantly correlated with the survival time after the blood was drawn (*p*-value = 0.036 and *p*-value = 0.026; Figure 4A,C) and the *p*-value was even reduced (*p*-value = 0.011) when combining *ESR1* variants in cfDNA and/or CTC gDNA in correlation with the survival time after the blood was drawn. Moreover, *ESR1* variants found in cfDNA significantly correlated with OS and survival time after the first diagnosis of metastasis (*p*-value = 0.0019 and *p*-value = 0.0033; Figure 4D,F). Univariate and multivariate analyses of histologic grade, tumor size at primary diagnosis and nodal status at primary diagnosis revealed *ESR1* variants as independent predictors of OS (univariate: *p*-value = 0.0094 HR = 9.2; multivariate: *p*-value = 0.038 HR = 18.68) and survival time after the first diagnosis of metastasis (univariate: *p*-value = 0.0092 HR = 5.9; multivariate: *p*-value = 0.019 HR = 117.19). In the cfDNA fraction, *BRCA2* variants also significantly correlated with survival time after blood was drawn and OS (*p*-value = 0.05 and *p*-value = 0.038; Figure 4B,E).

### 2.10. Cluster Analysis

**Figure 5:**
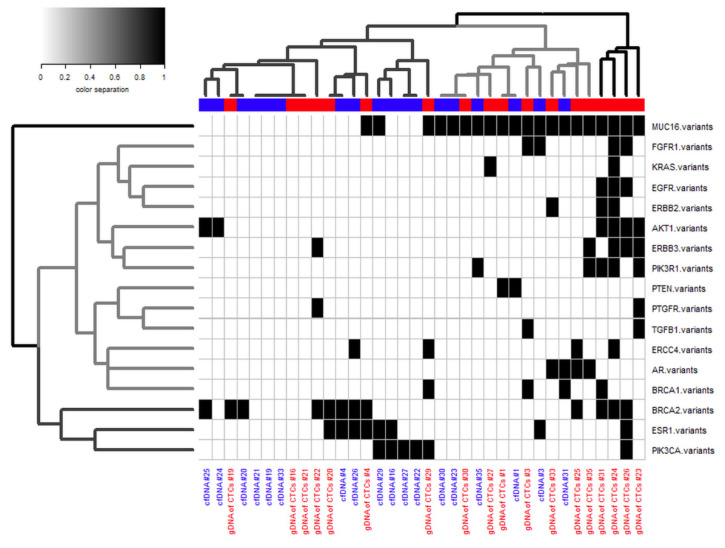
Heatmap plus hierarchical clustering summarizing the similarity of the samples. Samples showing at least one variant within the mentioned gene are marked in black. Hierarchical clustering was conducted according to Ward’s method with Euclidean distance.

The similarities between the matched cfDNA and CTC gDNA samples were summarized by hierarchical clustering (Figure 5). Dividing three clusters resulted in the aggregation of matched cfDNA and CTC gDNA samples from 11 patients within the same cluster (Figure 5 and Figure S2A), whereas setting 18 clusters (to examine whether the matched samples of the 18 patients clustered together) resulted in the aggregation of matched samples in only four cases (Figure S2B). Interestingly, one cluster, including *PIK3CA*, *ESR1* and *BRCA2*, was separated from the other two clusters, dividing the genes according to their similarity in the acquired data set (Figure 5).

## 3. Discussion

In this study, we established a workflow for matched cfDNA and CTC gDNA characterization from minimized blood volume via the isolation of CTC gDNA from mRNA-depleted CTC lysates and the isolation of cfDNA from CTC-depleted blood. In 94% of all patients, at least one variant was found post-filtering in either cfDNA or CTC gDNA. A fraction of 28% of the variants found in matched cfDNA and CTC gDNA samples was identical, showing the complementary nature of both analytes.

### 3.1. CTC gDNA Isolation and Library Preparation Was Successfully Established

In contrast to the established cfDNA isolation and sequencing protocol [16,24], a reliable variant analysis of CTC gDNA first had to be established. We aimed for a condensed workflow to isolate cfDNA, CTC gDNA and CTC mRNA from the same sample. Strong fragmentation of the DNA by the lysis buffer and the presence of inhibitory substances caused incompatibility with the tested WGA protocols. Moreover, the realization that a) 1 ng DNA can be sufficient as an input for targeted library preparation [23], b) that WGA was prone to inducing PCR artifacts and results in the allelic dropout of reads in the NGS evaluation [23] and c) the observation that the pyrosequencing results in the pre-experiments were more consistent without WGA, resulted in the exclusion of the WGA step in the final workflow. 

At the moment, most variant analysis protocols for CTCs use upstream WGA [10,11,12,13,14] and downstream ddPCR [18,25] or Sanger sequencing [9,12,13]. The establishment of a workflow for CTC gDNA variant analysis without WGA, but by targeted NGS with a parallel overexpression analysis of CTCs, was intriguingly difficult; however, to the best of our knowledge, we are the first to describe this workflow using only five ml of blood.

### 3.2. Sequencing Quality Parameteres Are Essential for Data Evalution

To guarantee the specificity of all called variants, we integrated UMIs and variantswere excluded if they showed less than five reads with different UMIs. Even though the average read count was doubled by a greater sequencing depth in CTC gDNA samples compared with the matched cfDNA samples, the absolute UMI count per CTC gDNA was still markedly lower. CTC gDNA samples exhibited, on average, a six-fold increase in reads per UMI, indicating a high duplicate read count.

The same observation was documented in the pre-experiments, where a greater sequencing depth (NextSeq run instead of MiSeq run) increased the reads/UMI. These findings suggest that very low amounts of input CTC gDNA are a limiting factor for the conversion of DNA fragments into library molecules and, thus, result in decreased library complexity.

The reduced count of unique sequencing reads (UMI depth) manifested in the limit of detection. Across all CTC gDNA samples, variants with a VAF of ≤10% were only called with a confidence of 90%, whereas, in the cfDNA samples, only the variants with VAF of ≤1% were called with a confidence of 90%. This demonstrates that the sensitivity to call variants with low allele frequencies is reduced due to the low complexity of CTC gDNA libraries. Therefore, we excluded all CTC gDNA samples where variants with a VAF of >15% were only obtained with a confidence of 90%. Even if this stringent criterion resulted in a reduction in the CTC gDNA samples included in further analysis (*n* = 27 to *n* = 18), it guaranteed callingcalled variants with at least >15% VAF. As a learning and in order to improve CTC gDNA sequencing in future, the input amount and complexity should be strengthened—for example, by improving gDNA yield in the isolation workflow. 

### 3.3. Characteristics of cfDNA and CTC gDNA Variants in Line with Previous Results

The number of detected variants in the CTC gDNA fraction was higher compared to the cfDNA variant number (127 versus 37). We suppose that this high CTC gDNA variant count is due to the CTC enrichment and is in contrast to the low cfDNA variant count, because tumor-derived cfDNA was not enriched from the cfDNA entirety. Consequently, the signal-to-noise ratio in the CTC gDNA fraction is better than in the cfDNA fraction.

The lower prevalence of likely pathogenic or pathogenic *PIK3CA* and *ESR1* variants in the CTC gDNA fraction may originate from an impaired number of low frequent, highly dynamic, tumor-derived variants (manifested by increased limit of detection; Table S2) compared to the potential germline variants (sequenced in the leukocyte contamination of the CTC-enriched fraction). In this context, it is important to discuss that the CTC gDNA variants with VAF between 5% and 10% might be the CTC variants in a background of leukocytes. Variants with VAF in this range were frequently located in the *MUC16* gene.

Interestingly, hierarchical clustering indicated a similar variant pattern in the cfDNA and CTC gDNA samples for the *PIK3CA*, *ESR1* and *BRCA2* genes. This observation is in line with the similar relevance of *ESR1* and *BRCA2* variants as prognostic factors, shown here and in other studies [26,27], and the comparable prevalence of *ESR1*, *PIK3CA* and *BRCA2* within the cohort. Furthermore, variants within these three genes are the most promising variants for therapy decision making [28,29,30,31,32,33]. 

In accordance with the prevalence of 40% of *PIK3CA* variants in the cfDNA of locally advanced BC patients [34], we also detected *PIK3CA* variants in 22%/11% of all patients in the cfDNA and CTC gDNA fraction. However, we could not reproduce the finding of 20% prevalence of *ERBB2* variants in cfDNA [34], but observed a prevalence of *ERBB2* variants in CTC gDNA of 28%. In contrast to a sensitivity of 68% for combined cfDNA and CTC variant analysis in metastatic prostate cancer patients [19], we observed that, in 94% of all 18 MBC patients, either cfDNA or CTC gDNA variants were found. This increased sensitivity is in accordance with the postulated advantage of multi-parametric approaches [35].

### 3.4. Variant Analysis in cfDNA and CTCs Is Complementary

The study was conducted to question the hypothesis that matched cfDNA and CTC gDNA variants reveal additive value. 

Only a few studies have compared cfDNA and CTC data thus far, but the blood volume needed for both analyses was additive and most of the isolation protocols required different preservative blood tubes for cfDNA and/or CTC storage. In BC, the total cfDNA level and CTC count were correlated with OS [14,36], cfDNA integrity was correlated with CTC presence [37] and specific ctDNA variants were correlated with CTC (cluster) prevalence [38]. cfDNA concentration or the presence of ctDNA outperformed CTC enumeration in terms of dynamic range, sensitivity, correlation with tumor burden, the earlier indication of response to chemotherapies and the earlier indication of impending relapse [2]. The cfDNA integrity had stronger diagnostic power than CTC counts [39], but improved sensitivity and specificity was proven when combining cfDNA and CTC counts as a diagnostic tool in non-MBC patients [34,39]. *SOX17* promotor methylation and *ESR1* methylation were highly concordant in ctDNA and CTCs [20,40] and cfDNA and CTCs showed overlapping mutation profiles [14,21], but cfDNA variant analysis detected more patients with *ESR1* variants [25] and detected more variants in general [14] than were identified by CTC variant evaluation. When studying cfDNA variants and CTC expression of the same 10 ml of blood, we described the additive value of both analytes [16]. 

In this study, 62% of the detected 37 cfDNA variants were recovered in the CTC gDNA fraction in 18 MBC patients. Consequently, 9% of all 164 detected variants in both fractions were unique to cfDNA, 63% of all variants were unique to CTC gDNA and 16% (29 variants) were shared in both fractions. The reported concordance of cfDNA and CTC gDNA variants is in accordance with the findings of Hodara et al., who compared cfDNA variants and CTC gDNA variants in 20 prostate cancer patients and reported a concordance of 13.8% [19]. In accordance with their results, we also found a highly reduced prevalence of *PIK3CA* variants in the CTC gDNA compared to cfDNA [19]. However, Kidess-Sigal et al. published contrasting results that reported a 91.3% concordance of *PIK3CA* variants in the cfDNA and CTC gDNA of advanced colorectal cancer patients [9]. These differences might be explained by the highly diverse variant analysis strategies, Sanger sequencing versus targeted PCR-based NGS. 

On average, the cfDNA variants with a mean VAF of 17% were not recovered in CTC gDNA, while the cfDNA variants that were recovered in CTC gDNA exhibited a mean VAF of 42%. Similarly, the unique CTC gDNA variants had a mean VAF of 20%, while the shared variants were prevalent, with a mean VAF of 48% in the CTC gDNA fraction. These data indicate that variants with VAF around 50%, potentially heterozygous germline variants, can easily be recovered in both fractions, whereas variants with lower VAF are difficult to detect in matched analytes. This might be explained by the lack of enrichment for tumor-derived cfDNA in contrast to the existing enrichment of CTCs. A limitation of this study is the lack of germline controls. Thus, we can only speculate whether specific variants might be tumor-derived or germline variants.

Most importantly, 84% of the detected variants were unique to one analyte, revealing the complementary nature of cfDNA and CTC gDNA, which has already been reported in other tumor entities [9,18,41,42] and BC [43]. Hierarchical clustering of the present study data revealed that matched cfDNA and CTC gDNA samples seemed to be similar in low-resolution clustering with three clusters, but the complementary nature of the matched samples became obvious in cluster analysis with higher resolution, dividing 18 different clusters. Therefore, and as postulated by Haber and Velculescu in 2014 [44], our findings confirmed the assumption that CTC and ctDNA are likely to be synergistic rather than strictly competitive.

## 4. Materials and Methods

### 4.1. Patient Population Characteristics and Eligibility Criteria

The study was conducted at the Department of Gynecology and Obstetrics, in collaboration with the Department of Medical Oncology, both at the University Hospital Essen, Germany and the Marienhospital Bottrop, Germany (for specimen recruitment) and also at QIAGEN GmbH, Hilden, Germany (for library preparation and sequencing analysis). In accordance with the Declaration of Helsinki, written informed consent was obtained from all participants at enrollment and specimens were collected using protocols approved by the Ethics Committee of the University Hospital of Essen (12-5265-BO). In total, blood samples from 28 MBC patients were studied between August 2015 and May 2019. The data of ten patients were excluded due to stringent exclusion criteria described below, resulting in a cohort of 18 MBC patients. All participants were ≥18 years, had Eastern Cooperative Oncology Group (ECOG) scores for a performance status of between zero and two, no severe, uncontrolled co-morbidities or medical conditions and no secondary malignancies. Prior neoadjuvant and adjuvant treatment, radiation, all kinds of surgical intervention or any other treatment of BC was permitted. MBC patients had estrogen (ER) and/or progesterone (PR) receptor-positive primary tumors (summarized as hormone receptor-positive (HR+)) and no ERBB2 overamplification (*n* = 15). Patients with ER-positive and/or PR-positive and HER2-negative metastases were also included if their ER, PR and HER2 status in the primary tumor was unknown (*n* = 3). All patients showed a progressive MBC when blood was drawn, evaluated by visual staging and according to the Response Evaluation Criteria in Solid Tumors (RECIST) [45]. Patient characteristics are listed in Table S1.

### 4.2. Sampling of Blood, Processing of Plasma and Enrichment of Circulating Tumor Cells

Samples of 2 × 9 ml EDTA blood were collected in S-Monovettes® (Sarstedt AG & Co), stored at 4 °C and were processed within 4 h after the blood was drawn. CTCs were isolated in duplicate from 5 ml of whole blood by positive immunomagnetic selection, targeting EpCAM, EGFR and HER2 (AdnaTest EMT-2/StemCell SelectTM, QIAGEN GmbH, Germany) [46]. The CTC-depleted blood remaining after positive immunomagnetic selection was centrifuged at 1841× g for 8 min, as previously described [16], and plasma was frozen at −80 °C. For other studies, the mRNA was isolated from the entire CTC lysates [46]. The supernatant remaining from the CTC lysates after incubation with the Oligo(dT)_25_ beads, called the mRNA-depleted CTC lysate, was stored at −80 °C.

### 4.3. Isolation and Quantification of cfDNA

cfDNA was isolated from ≥1 ml plasma from CTC-depleted blood. The plasma sample volume used ranged from 1.2–6.2 mL (mean 4.2 mL; maximized plasma volume available). cfDNA isolation was conducted by affinity-based binding to magnetic beads (QIAamp MinElute ccfDNA Kit, QIAGEN) and as described previously [16,47]. cfDNA yield was calculated for all fragments with a length between 100 and 700 bp (assessed using the Agilent Chip High Sensitivity DNA).

### 4.4. Isolation of gDNA from CTCs

The mRNA-depleted CTC lysates were pooled in cases where two lysates were available from the CTC isolation in duplicate (*n* = 16). The total volume of the mRNA-depleted CTC lysates was used to isolate the gDNA by a newly established workflow called the AllPrep DNA/RNA Nano Kit prototype (QIAGEN GmbH, Germany). Briefly, the lysates were mixed with 200 µL AdnaTest DNA Binding Buffer and 20 µL proteinase K and incubated for 10 min at 56 °C. After the addition of 200 µL ethanol, the whole mixture was applied to a QIAamp MinElute Spin Column. The membrane was washed using 500 µL AdnaTest DNA Wash Buffer A and 500 µL AdnaTest DNA Wash Buffer B. The membrane was then dried by centrifugation and the gDNA was eluted with 23 µL AdnaTest DNA Elution Buffer. 

### 4.5. Library Construction

cfDNA and CTC gDNA were not amplified prior to library preparation. The libraries were constructed with a customized QIAseq Targeted DNA Panel Kit (QIAGEN GmbH, Germany), targeting all exonic regions of *AKT1, AR, BRCA1, BRCA2, EGFR, ERBB2, ERBB3, ERCC4, ESR1, KRAS, FGFR1, MUC16, PIK3CA, PIK3R1, PTEN, PTGFR* and *TGFB1*. Library preparation using cfDNA was described in detail previously [16,47]. The input amount preferred for library preparation was in the range of 30–60 ng, but cfDNA samples with lower input were also included in the library preparation. The cfDNA samples had a mean input amount of 38 ng (range: 5–60 ng). 

The library preparation using the CTC gDNA was based on the protocol for cfDNA library preparation, but was adapted for some details, as described in the following. The gDNA was not quantified, but the entire eluate (20 µL) was used as the input. Enzymatic fragmentation, end-repair and A-addition were performed without the use of the FG solution and by the elongation of the incubation time at 32 °C to 24 min. The 5× volumes of the barcoded adapters, including the UMIs and sample-specific indices, were available for ligation to the gDNA in comparison to the cfDNA. The library preparation was then conducted without further modification as compared to the cfDNA protocol. 

### 4.6. Sequencing

The libraries were quantified by RT-qPCR and the quality was checked by Agilent Chip High Sensitivity DNA (Santa Clara) [47]. The libraries were diluted to 20nM (cfDNA)/10 nM (CTC gDNA). CTC gDNA libraries were first analyzed by paired-end sequencing on an Illumina MiSeq instrument using the MiSeq Reagent Nano Kit v2 for up to 1 million paired-end reads to check the desired dilution and equal distribution of all libraries within the pool. All pooled libraries were analyzed by paired-end sequencing on an Illumina NextSeq instrument using the NextSeq 500/550 High Output Kit v2.5, with 2 × 150 bp reads, using a custom sequencing primer (QIAseq A Read1 Primer).

### 4.7. Data Analysis/Bioinformatical Analysis

The bioinformatical analysis of the raw sequencing data of cfDNA and CTC gDNA was performed on the basis of the pipeline previously described [16], but some steps were modified. The sufficient sequencing quality of all samples was guaranteed by the exclusion of cfDNA libraries with less than 4 million read fragments, a UMI coverage lower than 400 and if less than 94% of the target region was covered with at least 5% of the mean UMI coverage. CTC gDNA libraries were excluded when the 90th percentile estimated minimum detectible allele fraction (LOD) was below 15%, which means that, in all included samples, the variants with over 15% variant allele frequency (VAF) were called with at least 90% probability. The input amount, library yield and sequencing quality parameters of each sample are summarized in Table S2. For analysis, we used the NGS Analysis service for QIAseq Targeted DNA Panels available at the QIAGEN GeneGlobe, which allowed for reliable variant identification based on UMI information. Ingenuity Variant Analysis (IVA; QIAGEN) was further used for the annotation, scoring, filtering and interpretation of the resulting variant files.

The IVA used five different filters for the exclusion of called variants. The confidence filter excluded all variants with an call quality below 20 and only included variants that passed the upstream pipeline filtering (primary data analysis in QIAGEN GeneGlobe using smCounter2). The upstream pipeline filtering used the following exclusion criteria: too many discordant read pairs, inside or flanked by homopolymer region, low coverage (fewer than 5 UMIs), variant in low complexity region (as defined by RepeatMasker), low base quality (mean < 22), too many genome reference mismatches in reads (default threshold is 6.5 per 100 bases), variants clustered immediately after the primer (possible enzyme initiation error), variant in simple repeat region (as defined by RepeatMasker), variant in simple tandem repeat region (as defined by Tandem Repeats Finder), strand bias and variant in micro-satellite region (as defined by RepeatMasker). Manually, all variant results in each patient were checked and excluded when the call quality was <20. Variants with a prevalence of 3% in the normal population were excluded, unless the variant had already been known to be a pathogenic common variant (common variant filter). The genetic analysis filter and the cancer driver variant filter were applied as described elsewhere [16], but the default setting responsible for the removal of all variants from the variant call format (vcf) group analysis output that were not detected in ≥ 50% of all studied cases was removed.

The calleded variants that were located in a polyQ stretch, and which were either L > Q or Q > L alterations, were excluded manually. It is important to mention that the variant nomenclature used in this publication refers to the transcript/protein variant with the longest amino acid sequence.

Original raw sequencing data are available at the European Nucleotide Archive under the study accession number PRJEB33921 and the sequencing quality parameters are listed in Table S2, while all obtained variants and corresponding allele frequencies are listed for each patient and each analyte in Table S3.

### 4.8. Statistical Analysis

Statistical analysis was performed by SPSS, version 11.5 (SPSS Inc., Chicago, IL, USA). Potential differences between the prevalence of variants within identical genes in both fractions were calculated by an exact two-sided Mann–Whitney U test. A Kaplan–Meier estimator and Cox regression models were used to assess survival after blood was drawn, first diagnosis (indicated as overall survival (OS)) or first diagnosis of metastasis. Survival curves were compared using the Log-Rank (Mantel–Cox) test. A Cox proportional hazards regression analysis was used to estimate the univariate and multivariate hazard ratios where appropriate, using R with the R libraries shiny, ggplot2 and survival (R version 3.6.1). Hierarchical clustering, according to Ward’s method with Euclidean distance, was conducted using R with the additional R libraries heatmap.plus and hmisc (R version 3.6.1). Significant values are indicated as asterisks (* ≙ *p*-value < 0.05;** ≙ *p*-value < 0.005) in Figure 2. Diagrams were computed with R (R version 3.6.1), OriginPro version 2019 (OriginLab Corporation) and Microsoft Excel (Microsoft Corporation), except the Venn diagram, which was produced with the online tool BioVenn [48]. The midpoints in the violin diagram indicate the median, the black box indicates data from the first to the third quartile, the whiskers indicate the area within 1.5 of the interquartile range and the violins extend to the full range of data.

## 5. Conclusions

A comprehensive analysis of cfDNA variants and variants in matched CTCs by targeted deep sequencing in HR+ HER2- MBC cases was successfully established from only 10 ml of blood. A direct comparison revealed that the variants in both liquid biopsy analytes complement each other. Although the number of patients harboring actionable variants has to be enhanced to prove these observations, it is advisable to assess both cfDNA and CTC gDNA variants in order to provide a more comprehensive genomic picture for personalized strategies in metastatic breast cancer in the future.

## Supplementary Materials

The following are available online at https://www.mdpi.com/2072-6694/12/5/1084/s1, Table S1: Patient characteristics, Table S2: Sequencing quality parameters for the cfDNA and CTC gDNA sequencing, Table S3: cfDNA and CTC gDNA variants and corresponding allele frequencies calleded after the described stringent variant filtering listed for all patients (*n* = 18), Figure S1: Variant detection using mRNA-depleted CTC lysates, Figure S2: Hierarchical clustering of the samples.

## Abbreviations

BC	Breast cancer
cfDNA	Cell-free DNA
ctDNA	Cell-free tumor DNA
CTCs	Circulating tumor cells
CTC gDNA	Genomic DNA of CTCs
ECOG	Eastern cooperative oncology group
ER	Estrogen receptor
HR+	Hormone receptor-positive
HER2-	Human epidermal growth factor receptor 2-negative
IVA	Ingenuity variant analysis
MBC	Metastatic breast cancer
NGS	Next-generation sequencing
OS	Overall survival
UMI	Unique molecular indices
PR	Progesterone receptor
RECIST	Response Evaluation Criteria in Solid Tumors
VAF	Variant allele frequency
Vcf	Variant call format
WGA	Whole-genome amplification

## List of Genes

AKT1	AKT serine/threonine kinase 1
AR	Androgen receptor
BRCA1	BRCA1 DNA repair associated
BRCA2	BRCA2 DNA repair associated
EGFR	Epidermal growth factor receptor
EpCAM	Epithelial cell adhesion molecule
ERCC4	ERCC excision repair 4, endonuclease noncatalytic subunit
ERBB2	Erb-b2 receptor tyrosine kinase 2 coding for the HER2 protein
ERBB3	Erb-b2 receptor tyrosine kinase 3
ESR1	Estrogen receptor 1
FGFR1	Fibroblast growth factor receptor 1
KRAS	KRAS proto-oncogene, gtpase
MUC16	Mucin 16, cell-surface associated
PIK3CA	Phosphatidylinositol-4,5-bisphosphate 3-kinase catalytic subunit alpha
PIK3R1	Phosphoinositide-3-kinase regulatory subunit 1
PTEN	Phosphatase and tensin homolog
PTGFR	Prostaglandin F receptor
SOX17	SRY-box transcription factor 17
TGFB1	Transforming growth factor beta 1
