# Supplementary material for: Multimodal Targeted Deep Sequencing of Circulating Tumor Cells and Matched Cell-Free DNA Provides a More Comprehensive Tool to Identify Therapeutic Targets in Metastatic Breast Cancer Patients
Source: Cancers (Basel). 2020 Apr 27;12(5):1084. doi: 10.3390/cancers12051084 (PMC7281124; doi:10.3390/cancers12051084)
Supplement: Supplementary file 1 [file cancers-12-01084-s001.zip › supplementary/Supplementary .docx]

Multimodal Targeted Deep Sequencing of Circulating Tumor Cells and Matched Cell-free DNA Provides A More Comprehensive Tool to Identify Therapeutic Targets in Metastatic Breast Cancer Patients

Corinna Keup, Markus Storbeck, Siegfried Hauch, Peter Hahn, Markus Sprenger-Haussels, Oliver Hoffmann, Rainer Kimmig and Sabine Kasimir-Bauer


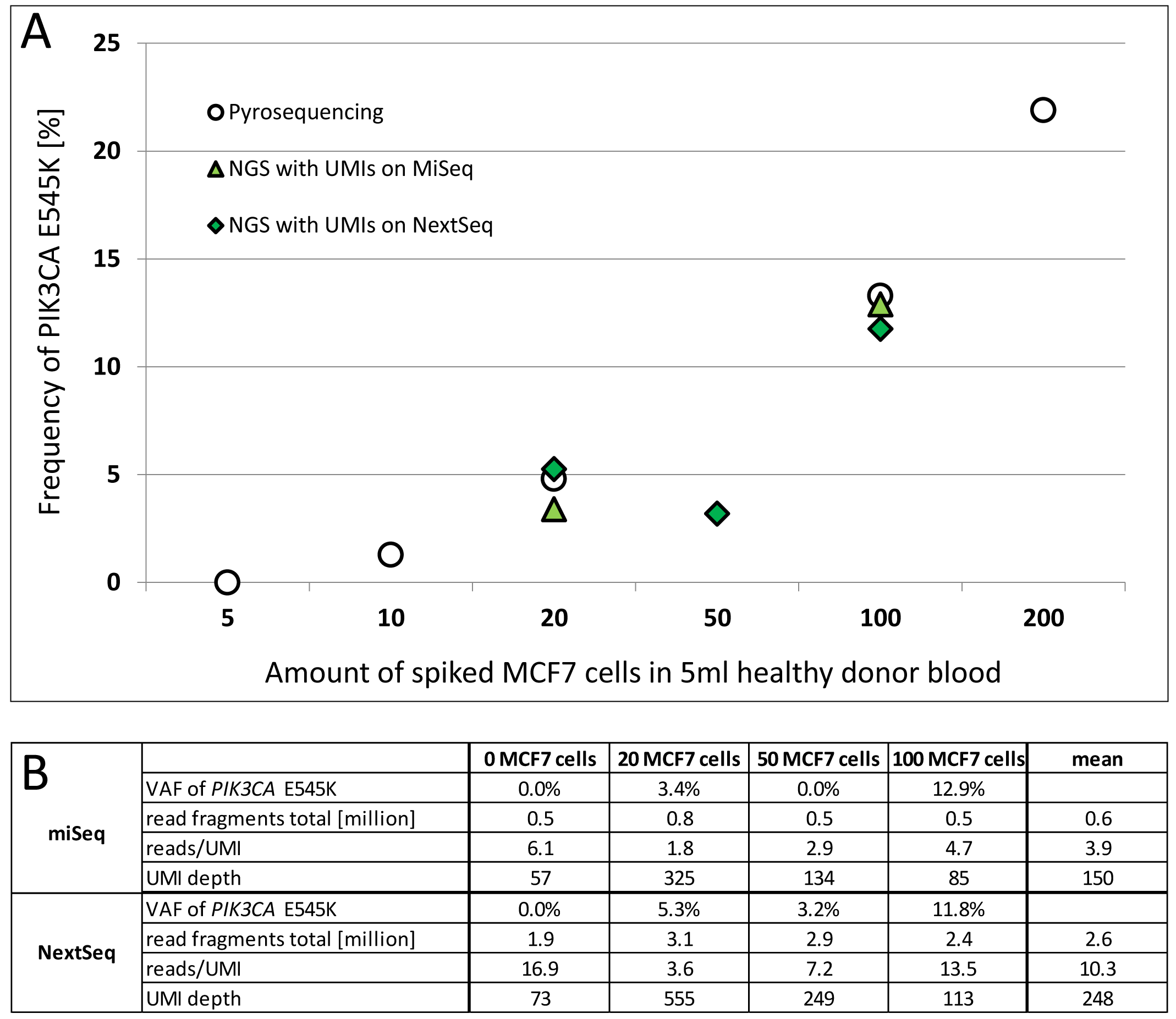


**Figure S1.** Variant detection using mRNA-depleted CTC lysates. Spike of MCF7 cells into blood of a healthy donor followed by the AllPrep DNA/RNA Nano prototype workflow (QIAGEN) without WGA, revealed the detection of the cell line specific variant *PIK3CA* E545K by pyrosequencing (circle) and targeted NGS (triangle in light green: customized QIAseq Targeted DNA Panel for Illumina with UMIs followed by a MiSeq run; diamond dark green: customized QIAseq Targeted DNA Panel for Illumina with UMIs followed by a NextSeq run). **A**) Plotted allele frequencies. **B**) Sequencing quality parameters.


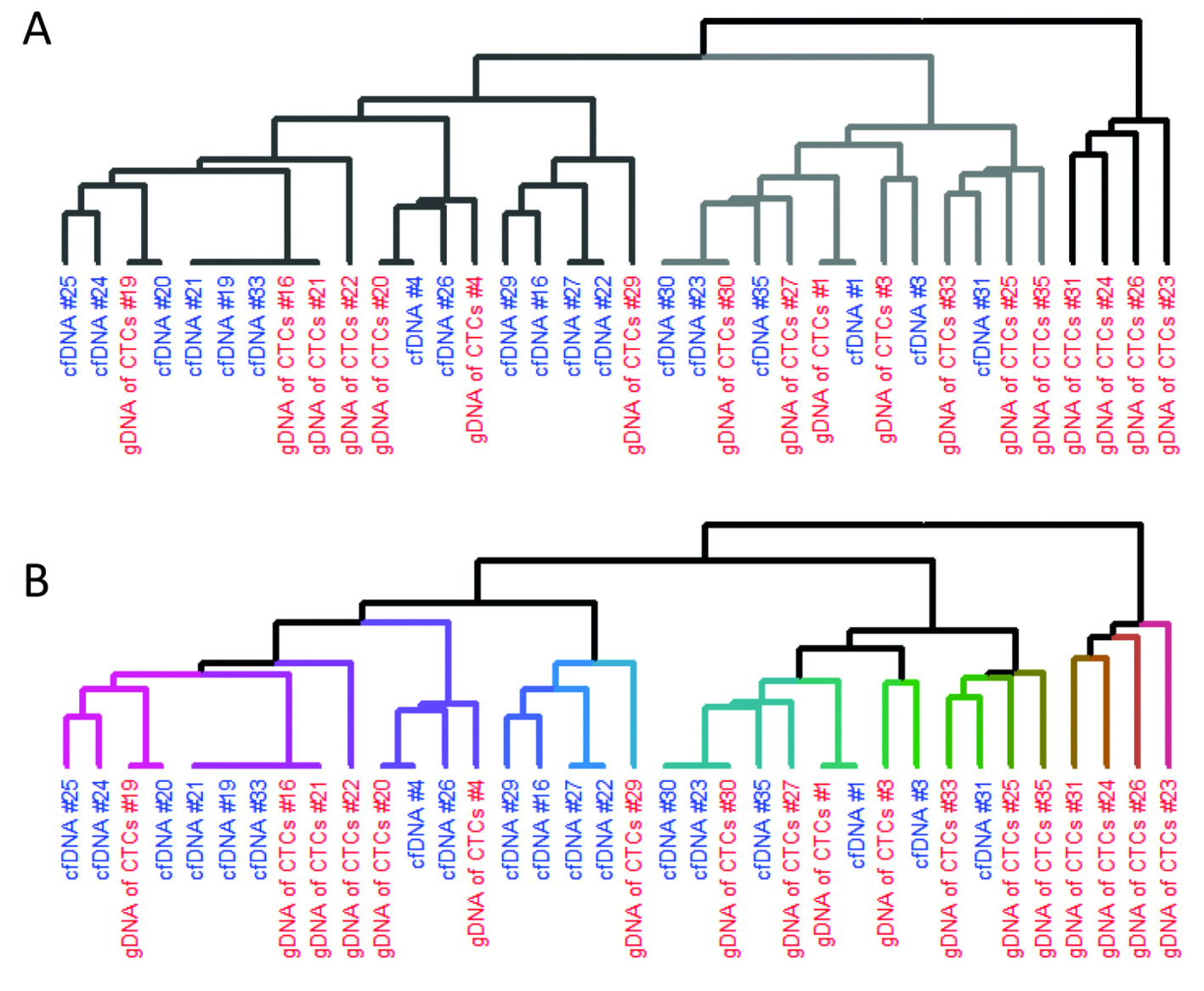


**Figure S2.** Hierarchical clustering of the samples. cfDNA samples marked in blue, CTC gDNA samples marked in red. **A**) Clustering dividing 3 subgroups of samples. **B**) Clustering dividing 18 subgroups of samples, as matched samples from 18 patients were used. However, only in four cases matched samples were clustered within the same subgroup.
